# Sludge degradation, nutrient removal and reduction of greenhouse gas emission by a *Chironomus*-*Azolla* wastewater treatment cascade

**DOI:** 10.1371/journal.pone.0301459

**Published:** 2024-05-28

**Authors:** Lisanne Hendriks, Tom V. van der Meer, Michiel H. S. Kraak, Piet F. M. Verdonschot, Alfons J. P. Smolders, Leon P. M. Lamers, Annelies J. Veraart

**Affiliations:** 1 Department of Ecology, Radboud Institute for Biological and Environmental Sciences, Radboud University, Nijmegen, The Netherlands; 2 Wageningen Environmental Research, Wageningen UR, Wageningen, The Netherlands; 3 Institute for Biodiversity and Ecosystem Dynamics, University of Amsterdam, Amsterdam, The Netherlands; 4 B-WARE Research Centre, Radboud University, Nijmegen, The Netherlands; Universiti Brunei Darussalam, BRUNEI DARUSSALAM

## Abstract

Wastewater treatment plants (WWTPs) are a point source of nutrients, emit greenhouse gases (GHGs), and produce large volumes of excess sludge. The use of aquatic organisms may be an alternative to the technical post-treatment of WWTP effluent, as they play an important role in nutrient dynamics and carbon balance in natural ecosystems. The aim of this study was therefore to assess the performance of an experimental wastewater-treatment cascade of bioturbating macroinvertebrates and floating plants in terms of sludge degradation, nutrient removal and lowering GHG emission. To this end, a full-factorial experiment was designed, using a recirculating cascade with a WWTP sludge compartment with or without bioturbating *Chironomus riparius* larvae, and an effluent container with or without the floating plant *Azolla filiculoides*, resulting in four treatments. To calculate the nitrogen (N), phosphorus (P) and carbon (C) mass balance of this system, the N, P and C concentrations in the effluent, biomass production, and sludge degradation, as well as the N, P and C content of all compartments in the cascade were measured during the 26-day experiment. The presence of *Chironomus* led to an increased sludge degradation of 44% compared to 25% in the control, a 1.4 times decreased transport of P from the sludge and a 2.4 times increased transport of N out of the sludge, either into *Chironomus* biomass or into the water column. Furthermore, *Chironomus* activity decreased methane emissions by 92%. The presence of *Azolla* resulted in a 15% lower P concentration in the effluent than in the control treatment, and a CO_2_ uptake of 1.13 kg ha^-1^ day^-1^. These additive effects of *Chironomus* and *Azolla* resulted in an almost two times higher sludge degradation, and an almost two times lower P concentration in the effluent. This is the first study that shows that a bio-based cascade can strongly reduce GHG and P emissions simultaneously during the combined polishing of wastewater sludge and effluent, benefitting from the additive effects of the presence of both macrophytes and invertebrates. In addition to the microbial based treatment steps already employed on WWTPs, the integration of higher organisms in the treatment process expands the WWTP based ecosystem and allows for the inclusion of macroinvertebrate and macrophyte mediated processes. Applying macroinvertebrate-plant cascades may therefore be a promising tool to tackle the present and future challenges of WWTPs.

## Introduction

About half of all wastewater produced globally is treated in wastewater treatment plants (WWTPs), but their efficiencies to degrade organic matter and to reduce nutrient concentrations vary substantially [1]. Hence, WWTPs remain a point source of organic and inorganic contaminants and nutrients, negatively impacting the discharge-receiving surface waters [2–5]. Moreover, during the treatment process, greenhouse gases (GHGs) are emitted, contributing to climate change [6], and large volumes of excess sludge are produced. The costs of processing and disposal of this excess sludge can make up to 60% of the total operational costs of a WWTP [7]. Therefore, new high-tech post-treatment technologies are being developed with higher nutrient removal rates [8, 9], but these are often expensive and energy demanding, contributing to global carbon emissions [10]. In response to these expensive and energy demanding technologies, the European Commission advocated that wastewater treatment should be cost-effective and energy neutral [11]. Moreover, as 48% of the global wastewater is not being treated at all, mostly in regions with limited sanitation infrastructure [1], these high-tech post-treatments may have a limited contribution to attaining the global Sustainable Development Goals (SDGs). Therefore, there is an urgent need for low-budget WWTP post-treatment techniques that further reduce the nutrient concentrations in the effluent, as well as the amount of produced sludge, while having a minimal GHG footprint. Moreover, such low-budget solutions may pave the way for application in regions still lacking any wastewater treatment.

As an alternative to technical solutions, we here argue that aquatic organisms have the potential to aid in sludge degradation and nutrient removal, as they also degrade organic matter and take up nutrients, especially nitrogen (N) and phosphorus (P), in their natural environment. Indeed, multiple species of macroinvertebrate collector-gatherers can feed on WWTP sludge, thereby affecting fluxes of nutrients and metals [12, 13]. They can also reduce GHG emissions from organically rich sediments, for example through burrowing, thereby oxygenating deeper layers and thus limiting methane (CH_4_) production and favouring CH_4_ oxidation [14]. A similar effect of benthic invertebrate bioturbation on WWTP sludge may be expected, because redox conditions in WWTP sludge are similar to those in organically enriched sediments. *Chironomus riparius* is a macroinvertebrate with a high sludge degradation capacity [15] occurring in high densities in organically enriched sediments [16], which makes it a suitable candidate for the treatment of wastewater. Macrophytes, including floating plants, can effectively remove nutrients from WWTP effluent [17, 18], and can affect GHG emissions positively or negatively by altering oxygen concentrations in the water column [19, 20]. Compared to other plants, *Azolla filiculoides* has a high nutrient removal potential (100% PO_4_^3-^ removal) and a high growth rate when grown on WWTP effluent [17]. Since it lives in symbiosis with a N fixing cyanobacterium, *Nostoc azollae*, it can overcome N limitation [21] and still remove P when N is limited. The produced biomass (doubling in 5 days [22]) may then be removed, permanently extracting the nutrients from the system and preventing nutrient discharge into the environment. Afterwards, this biomass can be sustainably post-processed. A cascaded setup may further allow for positive effects of both species, as well as facilitative interactions [23].

The aim of the present study was therefore to assess how well an experimental wastewater treatment cascade of bioturbating macroinvertebrates and floating plants is able to degrade sludge, remove N and P, and decrease GHG emission. To this end, an experiment was designed using a recirculating cascaded setup consisting of a wastewater treatment sludge compartment with or without bioturbating *Chironomus riparius* larvae, and an effluent container with or without the floating plant *Azolla filiculoides*. To calculate the N, P and carbon (C) mass balance of this system, we measured nutrient concentrations, biomass production, and sludge degradation, as well as the N, P and C content of all compartments in the cascade.

Bioturbating macroinvertebrates were hypothesized to promote the transfer of N and P into their own biomass and from the sludge into the overlying water, and to lower sludge CH_4_ emissions (Hypothesis (H)1). Furthermore, floating plants were expected to increase the transport of N and P from the water column into plant biomass, increase CO_2_ uptake, and decrease the emission of nitrous oxide (N_2_O) (H2). Lastly, it was hypothesized that the combination of bioturbating macroinvertebrates and floating plants would result in an increased transport of N and P into plant biomass, by invertebrate mobilisation of nutrients and subsequent uptake by plants, leading to a net lowering of N and P in the water column (H3a). As invertebrates and plants may affect GHG formation differently, the combination of organisms was expected to further limit GHG emissions from the two-compartment cascade (H3b).

## Material and methods

### Outline of the study

**Fig 1:**
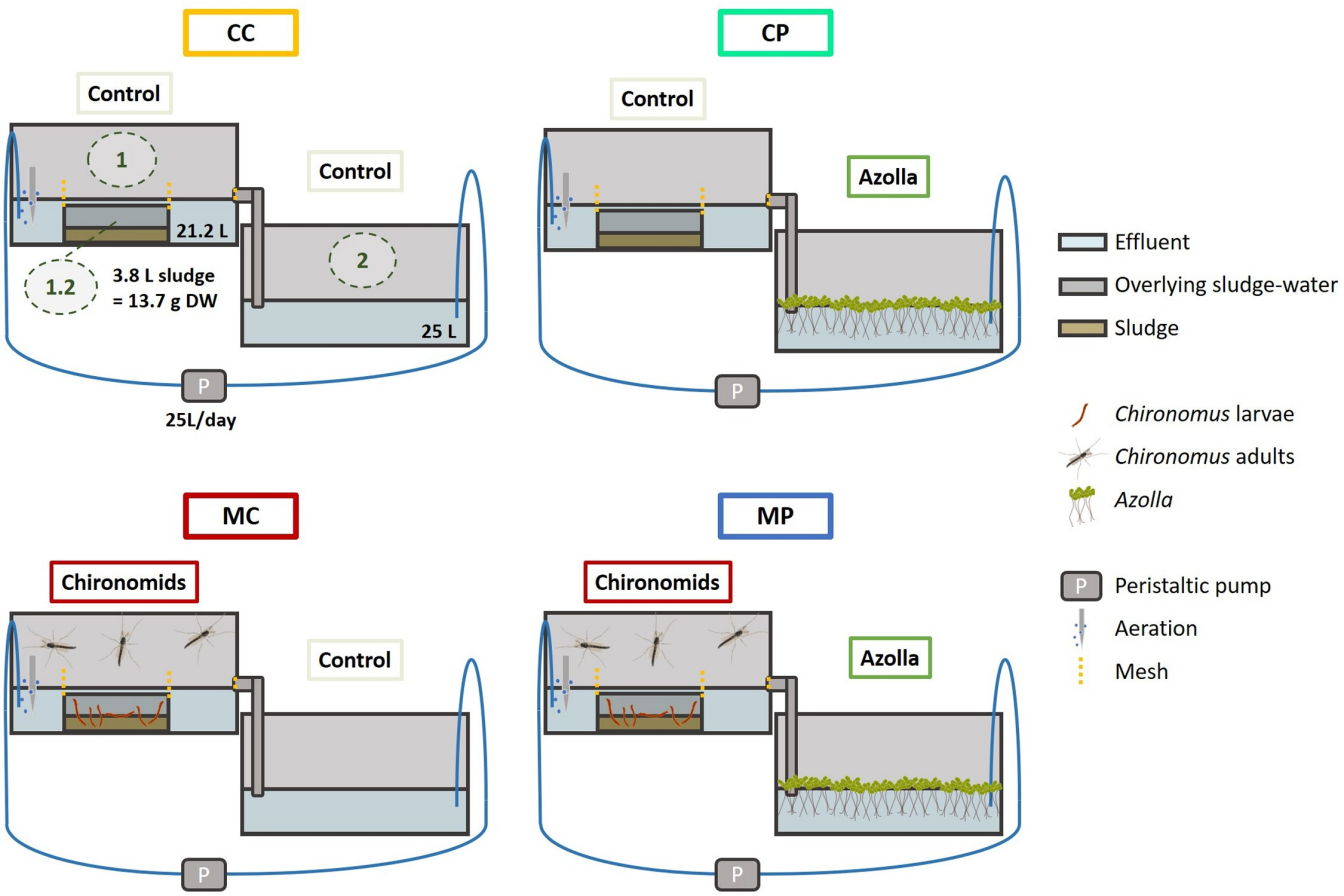
Schematic overview of the experimental setup. Cascades consisted of container 1 (1), filled with effluent and a sludge compartment (1.2), connected with a pipe to container 2 (2) which was also filled with effluent. A peristaltic pump (P) pumped the water from container 2 back into container 1. *Chironomus* larvae and egg ropes were added to container 1 of the MC and MP treatments, and *Azolla* was added to container 2 of the CP and MP treatments. To assess microbial sludge degradation, nutrient dynamics and GHG emissions, a control treatment without *Chironomus* and *Azolla* (CC) was also included in the setup.

To determine the effect of *Chironomus* and *Azolla* on sludge degradation, nutrient dynamics and GHG emission during the polishing of activated sludge and effluent from a WWTP, sixteen recirculating cascades were created, each consisting of two containers. In each of these cascades, the first container contained WWTP effluent and a small compartment with settled activated sludge, while the second container contained effluent (Fig 1). An overflow pipe connected the two containers, while water from the second container was pumped back into the first container by a peristaltic pump, creating a recirculating system. The full 2x2 experimental design consisted of four treatments: a *Chironomus*-*Azolla* (midge-plant; MP) treatment, a *Chironomus*-control (MC) treatment, a control-*Azolla* (CP) treatment and a control-control (CC) treatment, containing neither *Chironomus* nor *Azolla*. Ten-day old *Chironomus* larvae and egg ropes were added to the sludge compartment of the first container of the *Chironomus* containing treatments MC and MP, while *Azolla* was added to the second container of the *Azolla* containing treatments CP and MP. Each treatment consisted of four replicates. The experiment lasted for 26 days, during which dissolved nutrients and GHG emissions were measured twice a week, and emerging *Chironomus* adults and *Azolla* were harvested intermittently. At the end of the experiment, all biomass and remaining sludge were collected, weighed and C, N and P contents were determined.

### Methods

#### Collection of WWTP sludge and effluent

One day before the start of the experiment, 80 L activated sludge and 1000 L effluent were collected from the municipal wastewater treatment plant in Remmerden, The Netherlands, a UCT carrousel [24] with a 2100 m^3^ hour^-1^ hydraulic capacity that serves 46,000 households. In 2022, the sludge from the aeration tank had a (mean ± SD) dry weight of 3.75 ± 0.37 g L^-1^, and the effluent contained 390.6 ± 177.4 μmol L^-1^ N and 24.6 ± 8.1 μmol L^-1^ P.

#### Test organisms

*Chironomus riparius*. The non-biting midge *Chironomus riparius* (further referred to as *Chironomus*) is a common detritivorous macroinvertebrate, occurring in very high densities in organically enriched systems [16], where they construct burrows, thereby affecting sediment characteristics and nutrient dynamics [25].

*Chironomus* larvae and egg ropes originated from an in-house culture at Wageningen Environmental Research. *Chironomus* larvae were cultured in tanks containing a 3 cm sediment layer consisting of commercially available sand (63–210 μm), water column of Dutch Standard Water (DSW; deionized water 200 mg L^-1^ CaCl_2_•2H_2_O, 180 mg L^-1^ MgSO_4_•7H_2_O, 100 mg L^-1^ NaHCO_3_, and 20 mg L^-1^ KHCO_3_). *Chironomus* larvae were fed three times a week with a 9:1 Tetramin:Tetraphyll© (Tetrawerke, Germany) mixture. Half of the culture medium was renewed twice a month.

To obtain egg ropes and larvae, *Chironomus* adults were collected from the four culture tanks and placed in a flight cage, where they could mate and deposit their egg ropes in a small container containing sand and DSW. For the 10-day old larvae, these egg ropes were placed in freshly prepared culture tanks, where the eggs could hatch, and the larvae were collected after 12 days, as the mean hatching time was 2 days. These larvae were fed with the same food as the cultures.

*Azolla filiculoides*. *Azolla filiculoides* (further referred to as *Azolla*), is a floating plant occurring in eutrophic systems. *Azolla* can take up high amounts of carbon and nutrients resulting in a high maximal growth rate, outcompeting other plant species under eutrophic circumstances [17].

*Azolla* originated from an in-house culture at Radboud University, and was cultured in a greenhouse facility in large tubs (100 L) with a 16h/8h light dark cycle at 20.1 (16.2–25.2)°C. Before adding the plants to the experiment, they were transferred into smaller containers (40x60x10 cm) and grown on rainwater for two weeks, to ensure a low N, P and C content in the plants at the start of the experiment.

#### Experimental setup

The four replicates of each of the four treatments were distributed in a randomised block design to avoid confounding microclimatic effects in the greenhouse. Each cascade consisted of two polypropylene containers of 40x60x30 cm (l*w*h) with recirculating water. On the bottom of container 1 a smaller compartment (26.7x16.6x9.3 cm; 3.8 L) was placed containing WWTP sludge. To prevent detrimentally low oxygen concentrations for the *Chironomus* larvae, aeration was provided in two corners of container 1. An overflow pipe at a height of 12.5 cm allowed a maximum volume of 25 L. Excess water flowed into the second container, which was situated 15 cm lower. The outlet of this pipe was located 2 cm under the water level of container 2, which contained a volume of 25 L of effluent. The water from container 2 recirculated into container 1 via a Masterflex L/S peristaltic pump (Model No. 7528–30, Masterflex LLC, USA) equipped with a standard pump head (Model No. 7015–20), including high-performance precision platinum-cured silicone 4.88 mm tubing, with a hydraulic retention time (HRT) of 0.5 days (50 L day^-1^–35 mL min^-1^). To prevent algal growth in the tubing, all tubes were wrapped in aluminium foil. To prevent the *Chironomus* adults from escaping, containers 1 from the *Chironomus* containing treatments were covered with a mosquito net. Additionally, a mesh (1 mm mesh size) was attached to the sides of the sludge compartment, to prevent larvae from escaping from this compartment. Furthermore, all containers 1 and all containers 2 without plants (CC and MC treatments) were covered with white cloth to limit algae growth. The experiment was conducted in a greenhouse facility at Radboud University. To maintain a light/dark cycle of 16 h/8 h with sufficient light intensity, 400 W high-pressure sodium lamps (Hortilux-Schréder, The Netherlands) switched on when the natural daylight intensity was below 250 W m^-2^ during the 16h light period.

### Experimental procedures

#### Start of the experiment

One day before the start of the experiment WWTP sludge (3.8 L) was added to the sludge compartment of container 1, which was allowed to settle for 30 minutes. Thereafter, 21.2 L of effluent was carefully added to container 1, taking care not to disturb the settled sludge. The water in container 1 was high enough (12.5 cm) to also cover the 10 cm-high sludge compartment (Fig 1). To container 2, 25 L of effluent was added, resulting in a water level of 12.5 cm. To determine the initial dry weight per litre of sludge, as well as the N, P and C content of the dry mass and of the watery part of the sludge, six 2 L containers were filled with sludge, which was allowed to settle, after which water samples of water overlying the sludge were collected, excess water was removed, and all remaining sludge was collected. To determine initial nutrient concentrations of the effluent, a further six initial effluent water samples were collected. All samples were frozen at -20°C until analysis. At the start of the experiment, *Azolla* was introduced into container 2 of the *Azolla* containing treatments, covering 50% of the surface area. To the *Chironomus* containing treatments, 200 10-day old *Chironomus* larvae and 8 egg ropes were added to the sludge compartment of container 1. To determine the initial dry weight of both *Azolla* and *Chironomus* larvae, four additional plant batches (dry weight 5.6 ± 0.1 g), and three additional batches of 200 10-day old *Chironomus* larvae were collected from the culture, dried at 70°C and weighed. The experiment lasted for 26 days, and measurements of nutrient content and greenhouse gas fluxes were done biweekly.

#### Water quality measurements

To determine the dissolved nutrient concentrations (PO_4_^3-^, NH_4_^+^, NO_2_, NO_3_^-^, together NO_x_^-^) in the overlying water, filtered water samples were collected (pore size 0.12/0.18 μm, Rhizon SMS 10 cm, Rhizosphere Research, The Netherlands) of both containers from each replicate per treatment at the start of the experiment, before adding the organisms, and subsequently every 3 to 4 days. Samples were stored at –20°C until further analysis. The pH, temperature and dissolved O_2_ concentrations in the water column of each container were measured using a Portable Multi Meter (HQ2200, HACH, USA) with the appropriate probes (PHC20101, LDO1010). Due to practical constraints, filtered water samples to determine the dissolved organic carbon (DOC) and dissolved nitrogen (DN) content were only collected at the start of the experiment, after 12 and 19 days and at the end of the 26-day experiment. Samples were stored at 4°C until further analysis (see ‘nutrient analysis’).

#### Greenhouse gas fluxes

Diffusive greenhouse gas (CH_4_, CO_2_, N_2_O) emissions from all containers were measured at the start of the experiment, before adding *Chironomus* and *Azolla*, and subsequently every 3 to 4 days. Fluxes were measured using a Greenhouse Gas Analyser (G2508, Picarro, USA) connected to a transparent acrylic flux-chamber placed over the container. The edges of the flux-chamber were inserted 2 cm into the water column of the containers to seal the 10.4 dm^3^ headspace from the surrounding air. In each container, diffusive GHG fluxes were measured for 3 minutes, beginning at the moment that concentrations started to change. In-between the measurements, the chamber was aerated to return gas concentrations to atmospheric levels. To accurately calculate GHG fluxes, chamber air temperature was logged using HOBO Pendant® temperature data loggers (UA-001-64, Onset Computer Corporation, USA). Measurements were performed between 10:00 and 15:00 h.

#### Biomass collection

After 8, 15, 22 days and at the end of the 26-day experiment *Azolla* was harvested, reducing the plant coverage in each container to the original 50%. Exact coverage was ensured by creating a 100% coverage in a “harvesting container” of half the original container size, collecting the remaining *Azolla* from container 2, and returning all *Azolla* from the harvesting container into the original container 2. The collected biomass was dried at 70°C until completely dry. *Chironomus* adults started to appear after 8 days, which were collected by a customized vacuum-driven *Chironomus*-collector (adapted Turbo-Tiger, Princess™; S1 Fig). *Chironomus* adults floating on top of the water, exuviae and egg ropes were collected and counted as well. All *Chironomus* samples were stored at –20°C until further processing.

#### Ending the 26-day experiment

At the end of the 26-day experiment, all plants of the *Azolla* containing treatments were harvested. The overlying water from all containers, including the controls, was poured through a 38 μm sieve and all additional material (algae in control containers, *Chironomus* larvae that escaped from the sludge compartment in *Chironomus* containers, and *Azolla*-roots in *Azolla* containers) were collected. Thereafter, the overlying water of the sludge compartment was removed, and all remaining sludge was collected into 2 L pots. Sludge, *Chironomus* and additional accumulated leftover material were freeze dried. *Azolla* biomass was dried at 70°C until completely dry.

### Nutrient analysis

#### Dissolved nutrients

Concentrations of NH_4_^+^, NO_x_^-^ and PO_4_^3-^ in the filtered water samples were measured colorimetrically on an auto analyser (III, Seal Analytical, Norderstedt, Germany). NH_4_^+^ was determined using the Berthelot reaction (adapted NEN-EN-ISO 11732:2005), PO_4_^3-^ using an adapted ISO 15681–2:2003, and NO_x_^-^ according to an adapted version of NEN-EN-ISO 13395:1997. Total dissolved phosphorus (DP) and trace elements were measured in filtered acidified water (0.1 ml 10% nitric-acid) on an ICP-OES with a radial plasma observation, a V groove nebulizer and a cyclonic spray chamber (iCap 6300, Thermo Fisher Scientific, Bremen, Germany). DOC and DN concentrations were measured in rhizon-filtered samples on a total organic carbon analyser, using combustion catalytic oxidation at 680°C (TOC-L CPH/CPN analyser, Shimadzu). Each DOC and DN sample was measured twice.

#### N, P and C content in sludge, *Azolla* and *Chironomus*

Dried plant material was weighed and ground. Sludge, *Chironomus* adults, *Chironomus* larvae, exuviae and additional accumulated leftover material were freeze-dried at –90°C until completely dry. *Chironomus* larvae still present in the sludge at the end of the experiment were taken out from the freeze-dried sludge by hand, counted and weighed. *Azolla* and *Chironomus* larvae present in the leftover material were also manually separated and processed. Ground material (10 mg for sludge, 3 mg for other samples) was used to determine N and C concentrations using a CNS elemental analyser (Vario Micro Cube, Elementar, Langenselbold, Germany). To determine P and trace element concentrations, duplicate sample material (200 mg for *Azolla*, sludge and leftover material, 8–200 mg for *Chironomus* samples) was digested in Teflon vessels by adding 4 mL HNO_3_ (65%) and 1 mL H_2_O_2_ (35%). These samples were then heated in an Ethos One microwave (Milestone, Italy) for 20 minutes at 120°C. The digested samples were subsequently analysed on the previous-mentioned ICP-OES.

### Data analysis

#### Nutrient concentrations in water, and calculation of mass balances

The nutrient concentrations in the overlying water of both containers 1 and 2 were averaged per replicate and per day. DN concentrations were not measured one day before the start of the experiment. However, because DN was strongly related to DIN (NH_4_^+^ and NO_x_^-^ (R^2^ = 0.96)), we were able to estimate these missing DN values. To calculate the mass balances of N, P and C of all treatments at the start and the end of the experiment, the start and final amount of N, P and C was determined for each compartment in the system: water, sludge, *Chironomus*, *Azolla* and leftover material. To determine the start and final amount of N, P and C in the water, the concentrations in the water at day 0 and 26 were multiplied by the volume of the system (50 L). To determine the start and final amount of N, P and C in the sludge, *Chironomus*, *Azolla* and leftover material, the start and final dry weight of the sludge, the total harvested *Chironomus* and *Azolla*, and the leftover material with their respective nutrient contents were multiplied, using the following equation:

$$N_{tot}=\sum \left(\left[N_{w}\right]\times V_{w}\right)+\left(\left[N_{S}\right]\times DW_{S}\right)+\left(\left[N_{C}\right]\times DW_{C}\right)+\left(\left[N_{A}\right]\times DW_{A}\right)+\left(\left[N_{L}\right]\times DW_{L}\right)$$ (1)

Where *N*_*tot*_ is the total amount of N (mmol) in the cascade system, *N*_*W*_ (mmol L^-1^) and *V*_*W*_ (L) the N concentration and volume of the overlying water, *N*_*S*_, *N*_*C*_, *N*_*A*_, *N*_*L*_ (mmol g^-1^) the N content and *DW*_*S*_, *DW*_*C*_, *DW*_*A*_ and *DW*_*L*_ (g) the dry weight of respectively the sludge, the *Chironomus* biomass, the *Azolla* biomass, and the leftover material. The same formula was used for the mass balances of P and C.

#### Greenhouse gas fluxes

GHG fluxes (mg m^-2^ day^-1^) were calculated according to [26]. To convert the CH_4_ and N_2_O emissions into CO_2_ equivalents, multiplication factors were used of 27 and 273 respectively (global warming potential over a 100-year time frame [27]). Fluxes of CO_2_, CH_4_ and N_2_O below the minimum detectable flux (13.3, 2.4 and 151.9 mg CO_2_-eq m^-2^ day^-1^ for CO_2_, CH_4_ and N_2_O, respectively) were denoted as 0 [28, 29]. Measurements that were not useable due to sharp spikes in GHGs as a result of ebullition were counted as ebullition events. Cumulative GHG fluxes were calculated by the area under the curve divided by the 26-day period, expressing GHGs in CO_2_ equivalents.

#### Statistical analysis

Differences in DN, DP and DOC concentrations in the overlying water between the four treatments were assessed for every sampling date. Differences were assessed using one-way ANOVAs or non-parametric Kruskal-Wallis tests, followed by either a TukeyHSD or Dunn’s post-hoc test (with a Benjamini-Hochberg correction), depending on the occurrence of deviations from normality (Shapiro-Wilk test) and/or homogeneity (Levene’s test).

Differences in sludge DW, C, N, and P content between the treatments were assessed using a Welch One-Way analysis of means, with treatment as explanatory variable, followed by a Dunnett T3 post-hoc test to determine which treatments differed from each other. Since the data were normally distributed, but homogeneity of variance was not met, this is a robust and conservative method for a dataset with a small sample size [30, 31].

To determine if GHG emissions differed between experimental treatments, linear fixed models (*lmer*) were used, where both the day and the container were defined as random effects, and the presence of *Chironomus* larvae and *Azolla* were included as fixed effects. The complete model was compared to models only containing *Chironomus* or *Azolla* as fixed effects, and the best performing model was selected. Differences in CH_4_ emissions between container 1 and container 2 were also assessed using the same method, but with container as a fixed effect. As the CH_4_ emissions from container 2 and the effects of *Azolla* on CH_4_ emissions were non-significant, finally the effect of *Chironomus* on the CH_4_ emissions in container 1 was also assessed using an *lmer*, with the presence *Chironomus* as a fixed effect.

Between-treatment differences in ebullition observed in container 1 were assessed using Pearson’s chi square test for count data.

The effects of *Chironomus* and *Azolla* on the final mass balances of C, N and P were assessed by two-way multivariate ANOVAs (MANOVA), with C, N or P content in the sludge and in the overlying water as response variables, and the presence of *Chironomus* or *Azolla* as explanatory variables. N content was log transformed to meet the assumptions of (multi-variate) normality and homoscedasticity, which were tested using multivariate Shapiro-Wilk tests, and Box’s M tests respectively. When the results of the MANOVA were significant, two separate two-way ANOVAs were performed. This way we assessed whether significant changes were due to effects on either the sludge, the overlying water or both. All statistical analyses were performed in R 4.3.3 [32] using the packages lme4 [33], lmerTest [34] and emmeans [35] for linear fixed models, dunn.test [36] for Dunn’s tests, car [37] for Levene’s tests. The packages heplots [38] and mvnormtest [39] were used to check assumptions for the MANOVAs. For the creation of the figures, the ggplot2 [40] and ggpubr [41] packages were used.

## Results

### Experimental conditions

Water temperature increased over time from 20.6 ± 0.4°C to 24.3 ± 0.3°C (S2A Fig), and the pH ranged between 6.7 and 8.6 (mean = 7.6 ± 0.3; S2B Fig) during the 26-day experiment. Dissolved O_2_ concentrations were always above 6.2 mg L^-1^ (S2C Fig).

### Sludge degradation

**Fig 2:**
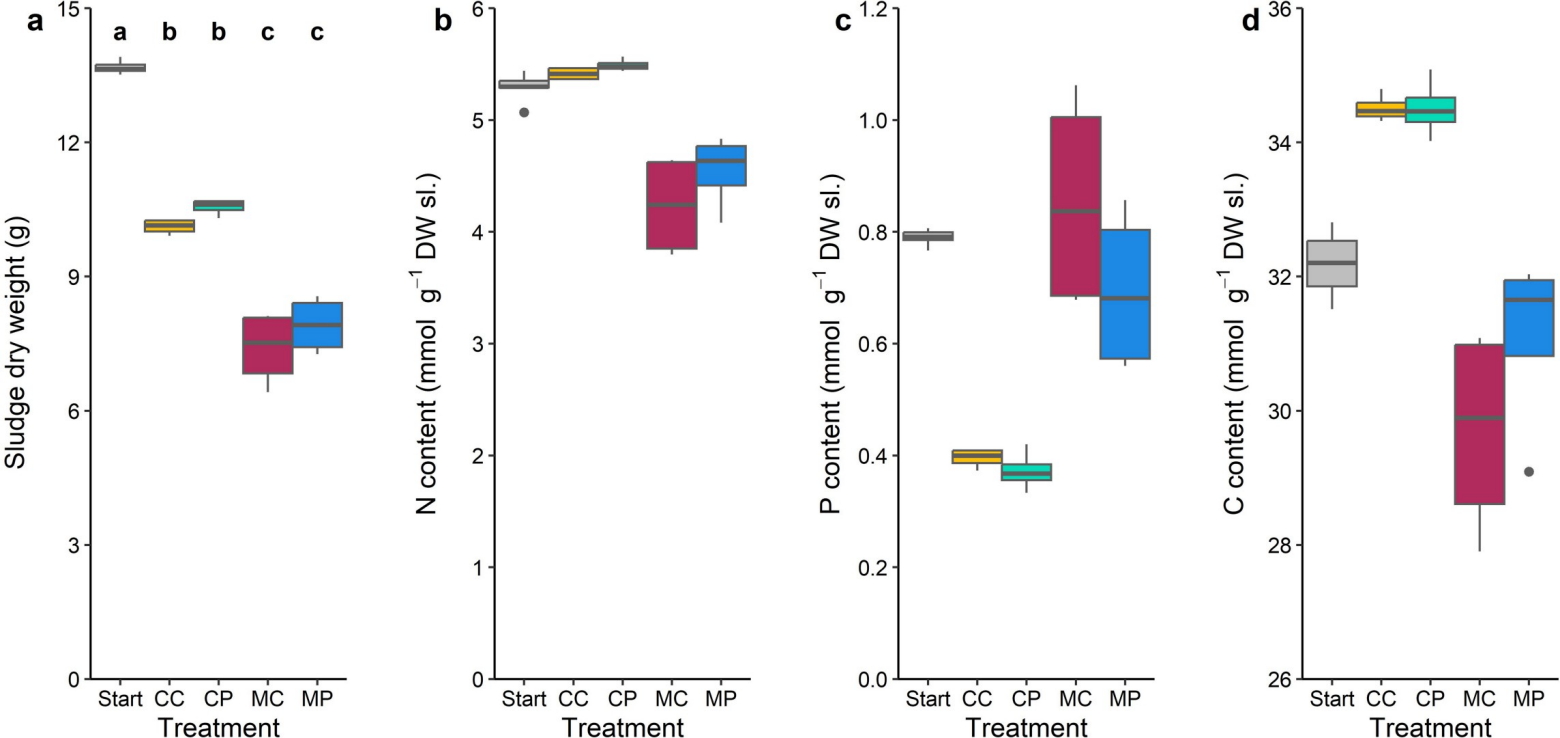
Sludge dry weight (a), N content (b), P content (c) and C content (d) of the initial sludge, and at the end of the 26-day experiment for the CC, CP, MC and MP treatments. Boxes show interquartile ranges, bold lines represent the median, whiskers indicate the lowest and highest values within a 1.5x interquartile range from the box, dots represent outliers.

Sludge dry weight at the start of the experiment was 13.7 g (SE = 0.06) per container and decreased during the 26-day experiment in all treatments (*F*_4,7_ = 367.08, *p* < 0.001; Fig 2A). Sludge dry weight at the end of the experiment in the *Chironomus* containing treatments (7.7 g, SE = 0.26) was significantly lower than in the treatments without *Chironomus* (10.3 g, SE = 0.10) (all *p* < 0.001), revealing that the presence of *Chironomus* caused a 1.8 times higher sludge degradation during the 26-day experiment (Fig 2A). Sludge N-content was affected by treatment (*F*_4,7_ = 13.23, *p* = 0.001; Fig 2B). Moreover, the N-content in the *Chironomus* containing treatments tended to be lower compared to the treatments without *Chironomus* (S1 Table). Sludge P-content also differed between treatments (*F*_4,7_ = 327.07, *p* < 0.001; Fig 2C). P-content in the treatments without *Chironomus* tended to be lower than in the treatments with *Chironomus* (S1 Table). Lastly, sludge C-content also differed between treatments (*F*_4,7_ = 31.07, *p* < 0.001; Fig 2D). Sludge C-content tended to be higher in treatments without *Chironomus* compared to treatments with *Chironomus* (S1 Table).

### Nutrient dynamics in the overlying water

**Fig 3:**
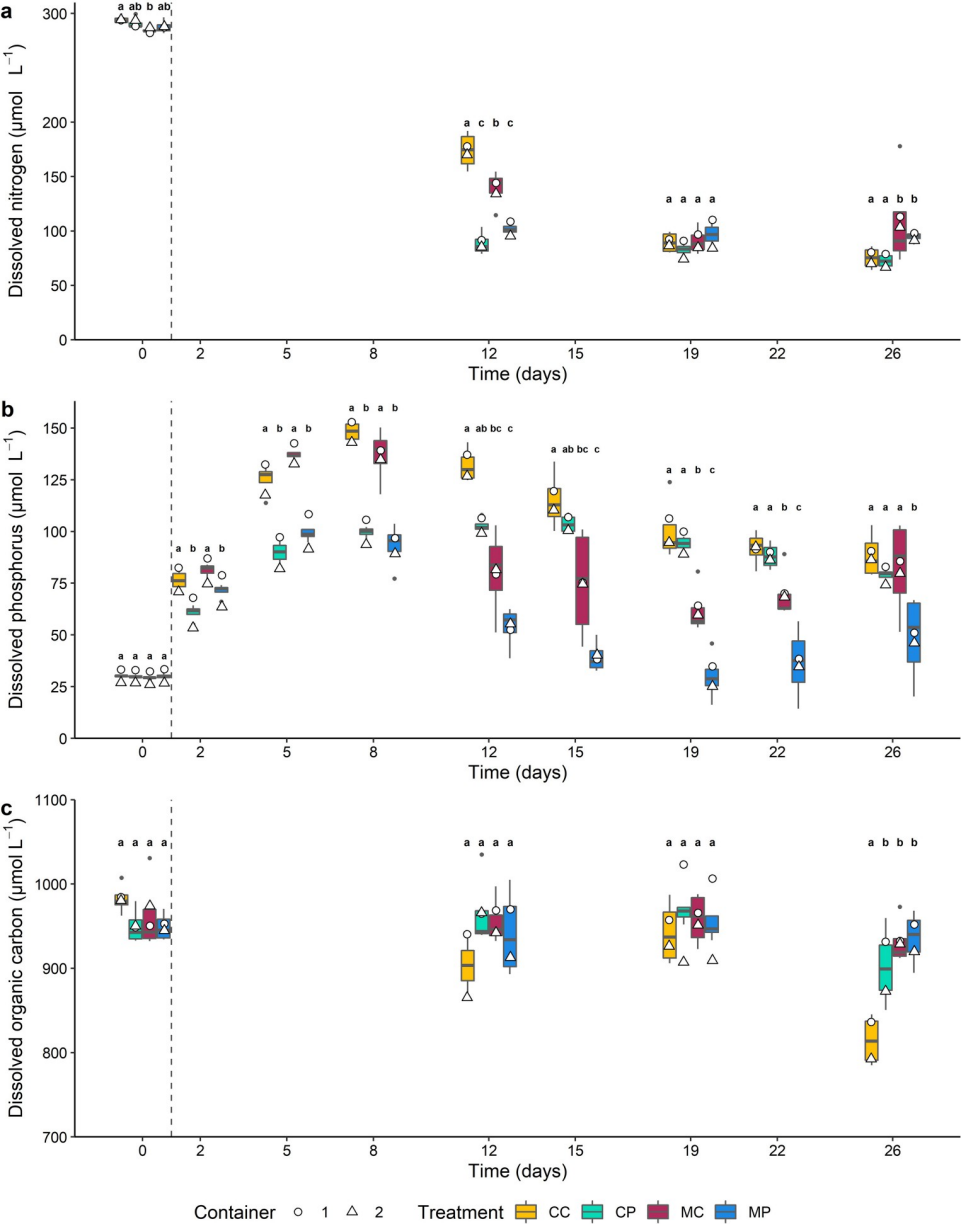
Dissolved nitrogen (a), dissolved phosphorus (b) and dissolved organic carbon (c) concentrations in the overlying water (μmol L^-1^) during the 26-day experiment for the CC, CP, MC and MP treatments. Boxes show interquartile ranges, bold lines represent the median, whiskers indicate the lowest and highest values within a 1.5x interquartile range from the box, dots represent outliers. White circles and triangles represent the average concentrations in respectively container 1 and container 2.

At the start of the experiment dissolved nitrogen (DN) concentrations in the water ranged between 287.9 ± 7.0 μmol L^-1^, and differed between treatments after 12 days (*F*_3,28_ = 59.0, *p* < 0.001). The *Azolla* containing treatments had a lower DN concentration than the *Chironomus* only treatment, which in turn was lower than the control treatment (all *p* < 0.001). At the end of the 26-day experiment, treatment still had an effect on the DN concentration (*Χ*^*2*^(3, *N* = 32) = 13.6, *p* = 0.003), since the DN concentration was lower in the control and CP treatment than in the *Chironomus* containing treatments (all *p* < 0.05; Fig 3A). NH_4_^+^ and NO_x_^-^ concentrations in the overlying water showed the same pattern (S3A and S3B Fig).

Dissolved phosphorus (DP) concentrations in the water were highest on day 8, and were affected by treatment (*F*_*3*,28_ = 60.2, *p* < 0.001). Both *Azolla* containing treatments (CP and MP) had a lower DP concentration than the treatments without *Azolla* (CC and MC; all *p* < 0.001). After 19 days, when the lowest mean DP concentrations were observed, DP concentration was still affected by treatment (*F*_3,28_ = 54.6, *p* < 0.001), but by then, the treatments without *Chironomus* (CC and CP) had a higher DP concentration than both *Chironomus* containing treatments (MC and MP), while in turn the MP treatment had a lower DP concentration than MC (all *p* < 0.001). Although at the end of the 26-day experiment, treatment still had an effect on DP concentration (*Χ*^*2*^(3, *N* = 32) = 16.2, *p* = 0.001; Fig 3B), only the treatment containing both organisms (MP) had a significantly lower DP concentration than the other treatments (all *p* < 0.02). PO_4_^3-^ concentrations in the overlying water showed the same pattern (S3C Fig).

After 12 days, approximately halfway the experiment, the DOC concentration in the overlying water was not affected by treatment, while at the end of the 26-day experiment the control treatment (CC) had a lower DOC concentration than the other treatments (*Χ*^*2*^(3, *N* = 32) = 15.6, *p* = 0.001, all *p* < 0.03; Fig 3C).

### Dynamics of *Chironomus* and *Azolla* biomass and NPC content

From the moment that the *Chironomus* adults started to emerge, the harvested adult *Chironomus* biomass increased over time (*t* = 3.06, *p* = 0.004), but did not differ between treatments (*t* = 0.76, *p* = 0.46; S4A Fig). Likewise, the presence of *Azolla* did not affect the biomass of any of the *Chironomus* life stages (S4B Fig). Furthermore, the *Chironomus* N, C, and P content did not differ between treatments, but P content in *Chironomus* adults increased over time (*t* = 3.25; *p* = 0.003) (S4C–S4E Fig). This resulted in an average P removal rate by *Chironomus* in the MC treatment of 410 ± 159 μmol P day^-1^ m^-2^ sludge and in the MP treatment of 276 ± 48 μmol P day^-1^ m^-2^ sludge. During the experiment the *Chironomus* adults produced on average 110.5 ± 23.6 and 148.8 ± 18.1 egg ropes per replicate in the MC and MP treatments, respectively.

Harvested *Azolla* biomass also increased over time (*t* = 7.54, *p* < 0.001), with an interaction effect of treatment (*t* = -2.61, *p* = 0.02), since the increase in *Azolla* biomass (S5A Fig) and the total produced *Azolla* biomass at the end of the experiment (S5B Fig) in the plant-only treatment (CP) was significantly higher than when *Chironomus* was also present (*F*_2,9_ = 143.2, *p* < 0.001). *Azolla* N and P contents decreased over time, while the C content increased (N: *t* = -2.16, *p* = 0.04; P: *t* = -13.31, *p* < 0.001; C: *t* = 3.67, *p* = 0.001), but the N and P contents did not differ between treatments (N: *t* = 1.97, *p* = 0.96; P: *t* = -0.19, *p* = 0.83). The C content of *Azolla* differed significantly between treatments (effect size = 0.5; *t* = 2.25, *p* = 0.03; S5C–S5E Fig). This resulted in an average P removal rate by *Azolla* in the CP treatment of 813 ± 23 μmol P day^-1^ m^-2^ *Azolla* cover and in the MP treatment of 669 ± 71 μmol P day^-1^ m^-2^ *Azolla* cover. The trace element and metal contents of Ca, Fe, Mn, Si, Zn and Cu in *Azolla* grown in the MP treatment were lower than in *Azolla* grown in the CP treatment. Concomitantly, concentrations of these elements and metals were lower in the overlying water and higher in the remaining sludge when *Chironomus* was also present (S2 Table).

### Reduction of greenhouse gas emissions

**Fig 4:**
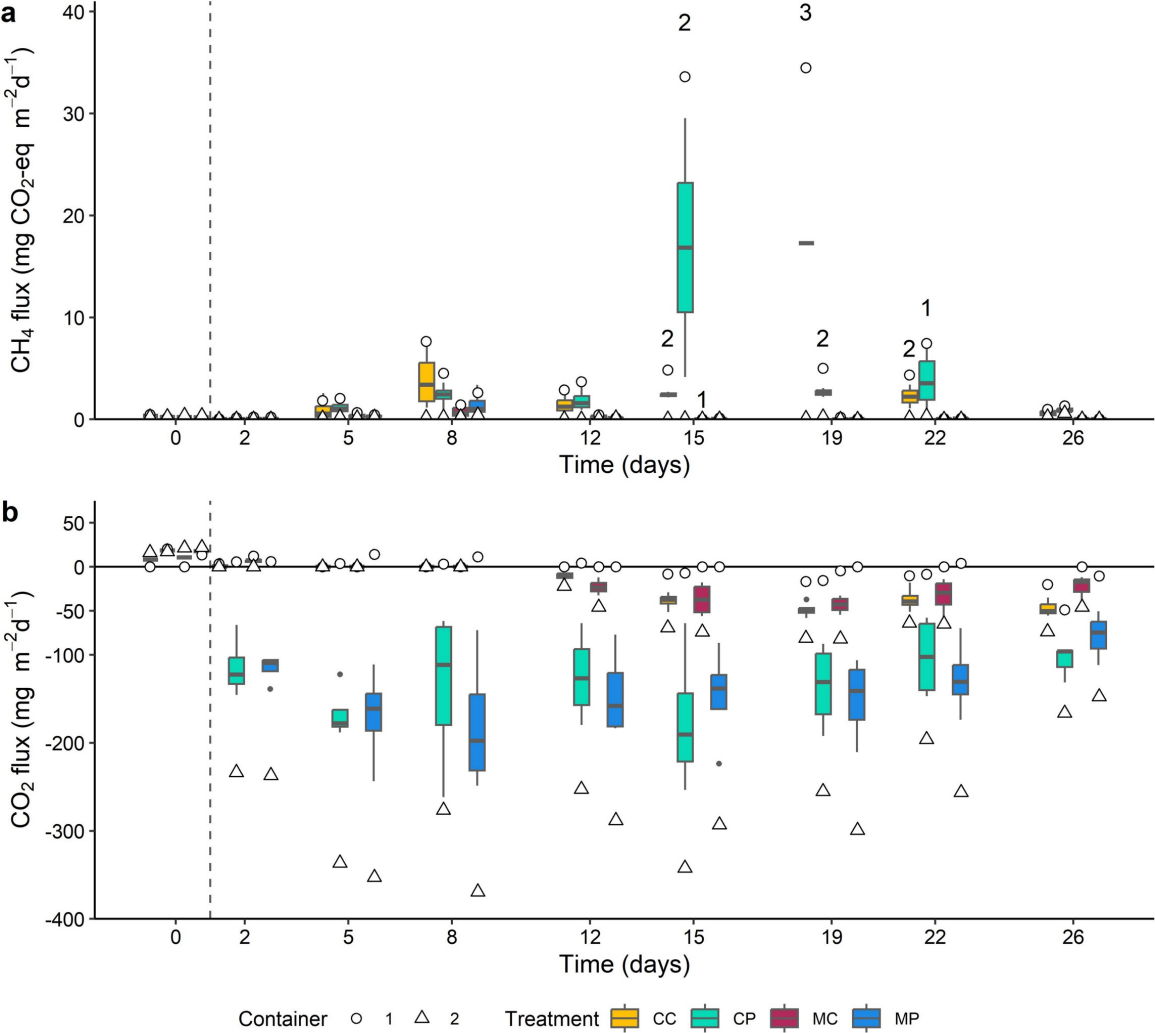
CH_4_ (a) and CO_2_ (b) fluxes for all treatments during the 26-day experimental period. Note the numbers given above some CH_4_ fluxes, which correspond to the number of flux measurements unusable due to ebullition in container 1. Boxes show interquartile ranges, bold lines represent the median, whiskers indicate the lowest and highest values within a 1.5x interquartile range from the box, dots represent outliers. White circles and triangles represent the average concentrations in respectively container 1 and container 2.

CH_4_ emissions were only observed in the sludge containing containers 1 (2.4 (SE = 0.6) mg CO_2_-eq m^-2^ day^-1^; *df* = 221.7, *t* = 4.1, *p* < 0.001), while CH_4_ fluxes in containers 2 did not exceed the minimum detection limit. The best fitting *lmer* model included only *Chironomus* as an explanatory variable, while *Azolla* did not increase the model fit, which was thus excluded. The emission of CH_4_ in containers 1 in the presence of *Chironomus* (0.384 (SE = 1.07) mg CO_2_-eq m^-2^ day^-1^) was significantly lower than in the absence of *Chironomus* (4.939 (SE = 1.14) mg CO_2_-eq m^-2^ day^-1^) (*df* = 14.0, *t* = 3.5, *p* = 0.004; Fig 4A), a reduction of 92%. Moreover, in total 13 out of 64 CH_4_ measurements were not usable because of ebullition in containers 1, which in 12 out of 13 cases appeared in treatments without *Chironomus* larvae (CC and CP; *X*^2^(1, *N* = 13) = 9.31, *p* = 0.002).

CO_2_ uptake occurred only in containers 2 (-148.2 (SE = 1.9) mg m^-2^ day^-1^, *df* = 225.56, *t* = -13.6, *p* < 0.001), whereas no uptake nor emission of CO_2_ was observed in the sludge containing containers 1 (1.2 (SE = 16.2) mg m^-2^ day^-1^, *df* = 18.5, *t* = 0.1, *p* = 0.94; Fig 4B). The best fitting *lmer* model included only *Azolla* as an explanatory variable, while *Chironomus* did not increase the model fit, which was thus excluded. *Azolla* presence significantly increased CO_2_ uptake in containers 2 (112.7 (SE = 7.4) mg m^-2^ day^-1^, *df* = 14.2, *t* = 15.2, *p* < 0.001), while treatments without *Azolla* did not show a significant CO_2_ emission, nor uptake (-14.6 (SE = 9.7) mg m^-2^ day^-1^, *df* = 6.3, *t*, = 1.5 *p* = 0.18).

During the entire 26-day experiment, N_2_O emissions did not exceed the minimum detectable flux in any of the treatments or containers.

When combining the cumulative contribution of the two GHGs, a net GHG-uptake (in CO_2_ equivalents) was observed in the *Azolla* containing treatments CP (-122.2 (SE = 12.0) mg CO_2_-eq m^-2^ day^-1^) and MP (-135.3 (SE = 10.4) mg CO_2_-eq m^-2^ day^-1^), whereas a very limited net effect on GHG emissions was observed for the CC (-13.2 (SE = 4.1) mg CO_2_-eq m^-2^ day^-1^) and MC (-17.5 (SE = 3.9) mg CO_2_-eq m^-2^ day^-1^) treatments. The net GHG emission of the CC and CP treatment are less accurate, because ebullition from these treatments was not taken into account.

### N, P and C mass balance

**Fig 5:**
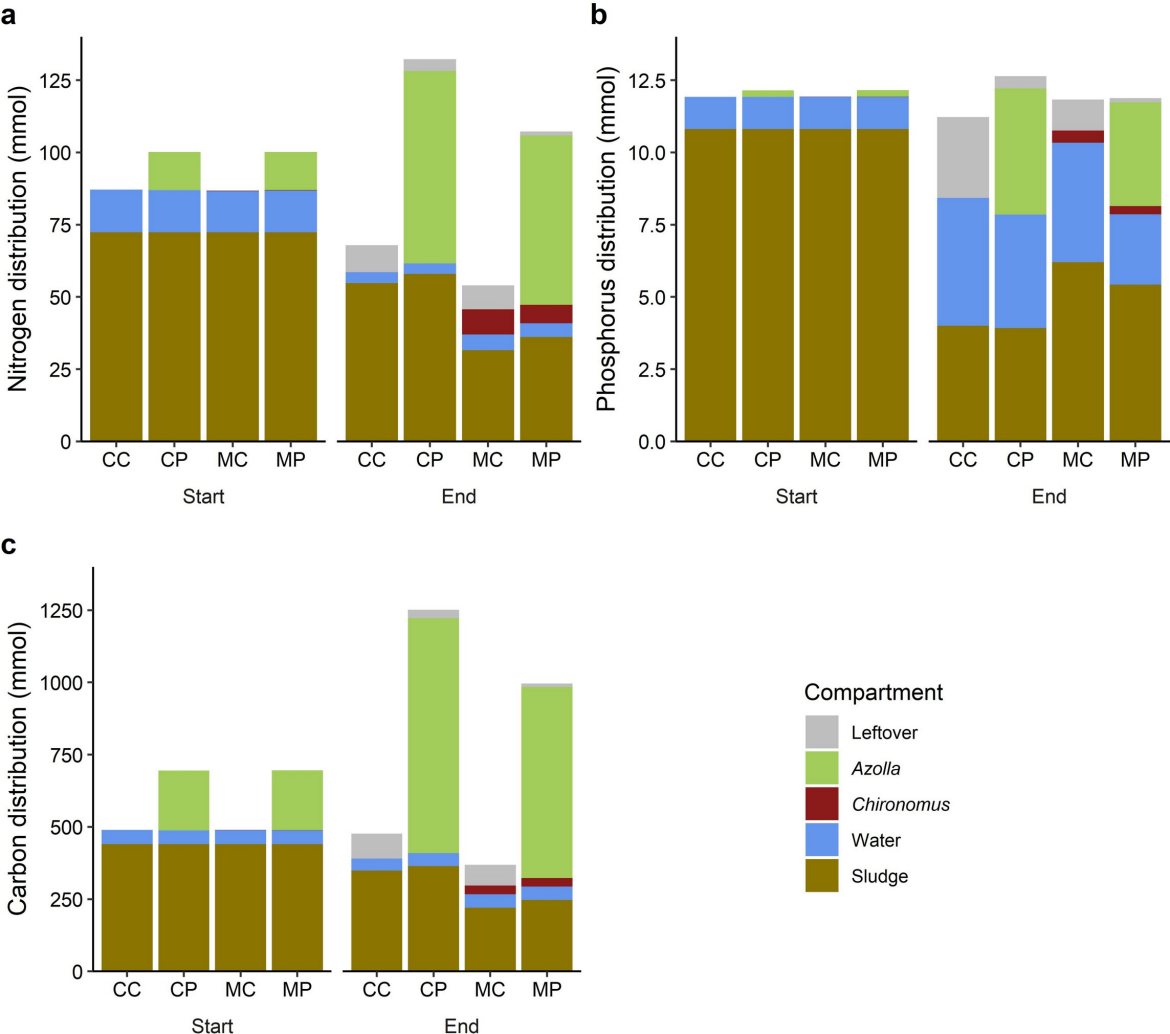
Mass balance of the treatment cascades for N (a), P (b) and C (c) in mmol at the start and the end of the 26-day experiment. Compartments include leftover material (grey), *Azolla* biomass (green), *Chironomus* biomass (red), overlying water (blue) and sludge mass (brown).

*Chironomus* affected the distribution of N between the sludge and the overlying water (*Pillai’s Trace* = 0.8, *F*_1,12_ = 27.6, *p* < 0.001; Fig 5A), whereas *Azolla* did not affect this distribution. The effect on the N distribution by *Chironomus* could largely be attributed to the lower final amount of N in the sludge in the presence of *Chironomus* larvae (*F*_1,12_ = 59.8, *p* < 0.001), which was partly due to the uptake by the larvae, whereas the amount of N in the overlying water was only marginally higher in the presence of *Chironomus* larvae (*F*_1,12_ = 7.4, *p* = 0.01).

The P distribution between the sludge and the overlying water was affected by both the presence of *Chironomus* (*Pillai’s Trace* = 0.9, *F*_1,12_ = 35.9, *p* < 0.001; Fig 5B) and *Azolla* (*Pillai’s Trace* = 0.8, *F*_1,12_ = 26.6, *p* < 0.001), which showed an interactive effect (*Pillai’s Trace* = 0.6, *F*_1,12_ = 11.0, *p* = 0.002). This was the result of the higher amount of P in the sludge in the presence of *Chironomus* larvae (*F*_1,12_ = 37.4, *p* < 0.001) and the lower amount of P in the overlying water in presence of the *Azolla* (*F*_1,12_ = 6.5, *p* = 0.025).

The C distribution in the cascades was also affected by both the presence of *Chironomus* (*Pillai’s Trace* = 0.9, *F*_1,12_ = 48.7, *p* < 0.001) and *Azolla* (*Pillai’s Trace* = 0.6, *F*_1,12_ = 7.1, *p* = 0.011). This was mostly due to the lower C amount in the sludge in the presence of *Chironomus* (*F*_1,12_ = 105.9, *p* < 0.001), and the lower amount of C in the water in the presence of *Azolla* (*F*_1,12_ = 7.90, *p* = 0.021), and/or the higher amount of C in the overlying water in the presence of *Chironomus* (*F*_1,12_ = 18.2, *p* = 0.001).

## Discussion

The present study assessed to what extent an experimental wastewater treatment cascade of *Chironomus* and *Azolla* was able to enhance sludge degradation, enhance nutrient removal and reduce GHG emissions. In line with our hypotheses, the presence of *Chironomus* led to increased sludge degradation, increased transport of N from the sludge into the overlying water and decreased CH_4_ emission (H1). However, contrary to our expectations, the transport of P from the sludge into the overlying water was limited in the presence of *Chironomus* larvae (H1). The presence of *Azolla* resulted in a lower TP concentration in the water column, and a higher uptake of CO_2_ as expected (H2). Interestingly, although the amount of P in the water column and GHG emission was indeed lowest in the treatment where both species were present (MP), this was not due to a facilitative effect where each organism altered water-conditions favourable to the other species, but rather due to the additive effects of the joint presence of both species (H3a and b).

### The effect of *Chironomus* on sludge degradation, nutrient dynamics and GHG emissions

*Chironomus* larvae almost doubled the sludge degradation compared to the control systems, which is in line with previous work on sludge degradation by *Chironomus* larvae [13]. *Chironomus* larvae could enhance sludge degradation even up to five times, when using higher densities of third instar larvae [15]. Furthermore, uptake of C and N by *Chironomus* larvae did not explain all C and N removed from the sludge. It is therefore likely that their bioturbation activity also stimulated the transport of C and N into the overlying water and subsequently to the atmosphere, leading to net C and N losses throughout the experiment. The release of C and N, for example in the form of CO_2_ or N_2_, from the sludge could have been caused by the bioturbation induced enhanced flux of oxygen into deeper layers of the sludge, which stimulated aerobic decomposition and coupled nitrification-denitrification [42]. P on the other hand, remained largely associated to the sludge. Apparently, the *Chironomus* larvae limited the transport of P from the sludge into the overlying water, most likely because the increased O_2_ concentration in the sludge resulted in effective binding of P to metal-oxides [43, 44]. The effect of bioturbation on the redistribution of P is highly dependent on the species-specific type of bioturbation, as well as on sediment characteristics. For instance, while the present study and [42] observed a reduced P concentration in the overlying water due to iron-coupled inactivation [25], reported a 21-fold increase in P concentration in overlying waters. This discrepancy may be explained by differences in OM contents, since the present study used sludge, which was very rich in organic matter, but also by the different type of burrows and bioturbation activity of the different benthic invertebrates. While *C*. *riparius*, used in our experiment, constructs J-shaped burrows, *C*. *plumosus*, used by [25], makes U-shaped burrows, which they ventilate, thereby transporting P rich pore water into the overlying water. During the first 8 days of the experiment, the DP concentration increased in all treatments, due to the initial release from the sludge when this started to degrade, but also due to the presence of a deep anoxic layer. No effects of *Chironomus* were observed during this period, likely due to their small size. Interestingly, after this initial peak, in the presence of *Chironomus* the DP concentration in the overlying water decreased until day 19, after which it started to increase again until the end of the experiment. Possibly, after initial bioturbation-mediated P-binding until day 19, sediment-binding sites were saturated, while at the same time P continued to be excreted due to feeding activity. This would indicate that bioturbation and feeding activity are antagonistic processes simultaneously mediated by *Chironomus*. As previously observed for metals [13], bioturbation by *Chironomus* larvae resulted in a greater change in the distribution of P and N in the system than the bioaccumulation within the organisms.

Bioturbation by *Chironomus* larvae also decreased CH_4_ emissions from the sludge by 92% and prevented CH_4_ ebullition. This emission-suppressing effect would be even stronger than presently calculated when considering the emitted GHGs by ebullition, which was especially happening when *Chironomus* was not present [26]. These observations indicate that in our experiment *Chironomus* burrows were likely an important CH_4_ oxidation site, and that their burrowing activity also prevented the built-up of GHGs as bubbles in the sludge [14]. *Chironomus* larvae did not affect CO_2_ emission, suggesting that the CO_2_ produced by their respiration was compensated for by reduced CO_2_ production of the microbial community.

*Chironomus* larvae can thus greatly affect the distribution of elements and processes in their benthic environment, having a positive effect on the three pillars of the present study: enhanced sludge degradation and nutrient removal, and reduced GHG emission.

### The effect of *Azolla* on nutrient dynamics and GHG emissions

During the first 12 days of the experiment, DN removal from the overlying water column was highest in the *Azolla* treatments. Although at the beginning of the experiment the NO_x_^-^ concentration in the water increased, this was compensated for by the removal of NH_4_^+^, pointing at nitrification rather than plant uptake as main NH_4_^+^ removing pathway. From day 8 onward, NO_x_^-^ concentrations in the water decreased, which proceeded faster in the presence of *Azolla*, suggesting either denitrification or NO_x_^-^ uptake. Even though earlier work on *Azolla* grown on wastewater effluent suggested that it did not decrease NO_x_^-^ concentrations, and therefore total N concentrations remained rather high [17], in the present cascade N concentrations in the water column were further reduced when *Azolla* was present. Besides NH_4_^+^ and NO_x_^-^ uptake, *Azolla* likely also fixed N from the atmosphere, as shown by the overall nitrogen balance, where the final amount of N exceeded the initial amount when *Azolla* was present. Although N fixation during NH_4_^+^ abundance may seem counterintuitive, this has been previously observed in the *Azolla*-cyanobacteria symbiosis. In other experiments, the N content in *Azolla* was more related to N fixation from the atmosphere than to N assimilation from NH_4_^+^ [45]. P uptake by *Azolla* removed DP from the overlying water column. Removal rates (669–813 μmol P day^-1^ m^-2^ *Azolla* cover) were in the same range as those reported for duckweed (450–2400 μmol P day^-1^ m^-2^ plant cover [46]), but lower than *Azolla*-mediated P removal observed in other studies (745–1100 μmol P day^-1^ m^-2^ *Azolla* cover [47]). This is likely because *Azolla* growth and P uptake in the treatment including *Chironomus* was limited by the low concentrations of trace elements in the water column, due to their increased binding to the sludge as a result of the *Chironomus* activity. Nonetheless, even though initial removal of N and P by *Azolla* was high, the final concentrations of these nutrients did not differ from those in the control treatments. Possibly, after 12 days, filamentous algae growing in control treatments started to affect the N and P dynamics and balance, since these algae are also known for their high N and P removal potential [48]. The presence of *Azolla*, however, prevented the growth of algae by blocking light penetration into the water column.

*Azolla* presence drastically reduced GHG emission. No CH_4_ was emitted from the *Azolla* containers, as was also observed in previous studies on hydroponically grown *Azolla* [17]. Additionally, even when CH_4_ would have been produced, harvesting the *Azolla* biomass limited the formation of a reaeration barrier, and thus O_2_ levels decreased only slightly. Moreover, *Azolla* captured high amounts of C, reflected by high CO_2_ uptake (1.13 kg ha.^-1^ day^-1^; Fig 4B), and subsequent C incorporation into their biomass. Under optimal growth conditions, this uptake might even be up to five times higher, up to 5800 mg m^-2^ day^-1^ [49].

As *Azolla* increased nutrient removal from the effluent and sequestered high amounts of GHGs, this plant may be a suitable option to use in a WWTP polishing cascade.

### Combined effects of *Azolla* and *Chironomus* on nutrient balances and GHG budget

No facilitative interactions were observed between *Chironomus* and *Azolla* regarding growth, which contrasts the findings of [23], who observed increased growth of *Azolla* when *Tubifex* worms were present in a preceding compartment in a comparable experimental cascade. This was attributed to a lowered pH of 4 and increased Fe water concentrations due to *Tubifex* sludge consumption. In our experiment, however, no effect of *Chironomus* activity on pH was observed, and the pH remained therefore relatively high in the *Azolla* containers, thereby possibly limiting *Azolla* growth [23]. *Azolla* sequestered less N, P and C in the presence of *Chironomus*, likely due to the lower growth rate of *Azolla*. Yet, the sludge P binding due to *Chironomus* activity compensated for this, resulting in the lowest overlying water P concentration in the presence of both *Chironomus* and *Azolla*. Contrasting to this additive effect of *Chironomus* and *Azolla* on the P distribution, *Chironomus* larvae stimulated the transfer of C from the sludge into the overlying water, whereas the *Azolla* removed C from the water column. Hence, *Chironomus* larvae and *Azolla* both affected specific GHGs at specific places in the cascade, and therefore also showed additive effects in the overall GHG dynamics, resulting in the highest carbon sequestration when they were both present. Therefore, the combination of both species, despite present in different compartments, resulted in the largest removal of P and N from the overlying water, as well as the largest GHG reduction through their additive, but not facilitative, effects on the P and N distribution in the system.

### Implications and challenges for future wastewater treatment

Our *Chironomus*-*Azolla* treatment cascade was able to efficiently redistribute the nutrients present in the experimental wastewater system. The N and P in the original wastewater remained either associated with the sludge or were incorporated into organism biomass, limiting the amount of nutrients present in the overlying effluent. These lower effluent nutrient concentrations were in concord with lower amounts of remaining sludge and lower GHG emissions, thus tackling three urgent challenges of WWTP operators: limiting excess sludge production and lowering GHG emissions and nutrient rich effluent discharges into surface waters, which are key for future proof WWTPs. Moreover, the remaining sludge contained a higher amount of P, which makes it more suitable to extract P to use it as a fertilizer, which is in line with the stated EU proposals [11].

Our experimental setup was primarily focused on assessing the effects of *Chironomus* and *Azolla* on the N, P and C dynamics, and the joint presence of the two species resulted indeed in the highest P removal from the water column, with the P concentration being almost two times lower than in the control treatment. Nonetheless, the final P concentration in the water was higher than at the start of the experiment. Hence, to increase the effectivity of the cascade, the dimensions should be adjusted to allow for a larger surface area of *Azolla* to take up the nutrients released during *Chironomus* sludge degradation.

To achieve a well-functioning real-life treatment cascade, the next focus should be on how to scale up these processes, both in time and space. Our experiment was performed under favourable conditions for the organisms, at 20–24°C with 16 hours of daylight, but in practice temperatures and light conditions may be less optimal during winter periods at more northern locations. Nonetheless, both *Azolla* and *Chironomus* can grow and reproduce at 4 and 14°C, respectively [22, 50], but under these conditions their growth rates are lower. Increasing light and temperature might then be an option, although this would increase costs and GHG emissions. Optimizing growth conditions for *Azolla* could lead to a P extraction of 1100 μmol P day^-1^ m^-2^ *Azolla* cover [47]. Furthermore, the larval density [51] and the harvesting rate of *Azolla* would also affect the efficiency of nutrient removal and sludge degradation and should be a focus of future chronic multi-generational experiments.

The proposed treatment does not require high-tech nor expensive equipment and may therefore be suitable to complement conventional wastewater treatment, especially at locations lacking the infrastructure to apply such high-tech wastewater treatment techniques. Depending on climate, water quality and sludge composition, other species combinations may be more, or less efficient in sludge degradation [15], assimilation of nutrients or GHG emission reduction [17]. The processes described here with *Chironomus* and *Azolla* may therefore be replicated in other climates using local species. As an alternative for *Azolla*, phytoplankton could be used to remove nutrients and contaminants from WWTP effluent [52], and the use of macroalgae is gaining attention as well [48]. The growth of filamentous algae in our experiment did indeed show that the employment of macroalgae could be a suitable option. Moreover, algae-filter feeder cascades have already been applied successfully on an experimental scale [53].

The harvested *Chironomus* and *Azolla* biomass may be used as a resource for novel products, but in choosing the most appropriate application, the contaminant concentrations should be considered. For instance, *Azolla* and other floating plants are already used as feed, renewable fuels and biofertilizer [54]. To limit risks associated to bioaccumulation of contaminants [55], non-food applications are preferred. Bioaccumulation of contaminants in sludge-grown *Chironomus* seems to be limited, but nonetheless contaminant concentrations did sometimes exceed allowable levels for feed and foodstuff [13].

## Conclusions

There is an urgent need for low-budget WWTP post-treatment polishing techniques that further reduce the nutrient concentrations in the effluent, as well as the amount of produced sludge, while having a minimal GHG footprint. Here, we showed for the first time that a *Chironomus-Azolla* treatment cascade can indeed reduce P and N concentrations in wastewater treatment effluent and degrade wastewater treatment sludge, while having a minimal GHG footprint and even showing GHG sequestration. Effects of *Chironomus* and *Azolla* on greenhouse gas emission reduction and nutrient removal were additive, highlighting the benefit of a cascaded two-species system. Thus, applying cascades of organisms in wastewater treatment may be a promising tool in conforming to new EU proposed guidelines for wastewater treatment and could lead to the design of low-cost, low-tech, widely applicable treatment systems.

## Supporting information

S1 Fig Adult Chironomid collection device. 1: Suction hose that is aimed at adult Chironomid. 2: Collection chamber and mesh bag, mesh bag can quickly be closed after vacuuming the Chironomids. 3: Tubing with valves to allow for the adjustment of suction power. 4: Vacuum device (Princess Turbotiger).
(TIF)

S2 Fig Temperature (°C) (a), pH (b) and dissolved O2 concentration (mg L-1) (c) in the overlying water during the 26-day experiment for the CC, CP, MC and MP treatments. Boxes show interquartile ranges, bold lines represent the median, whiskers indicate the lowest and highest values within a 1.5x interquartile range from the box, dots represent outliers. White circles and triangles represent the average concentrations in respectively container 1 and container 2.
(TIF)

S3 Fig Dissolved NH4+ (a), dissolved NOx- (b) and dissolved PO43- (c) concentrations in the overlying water (μmol L-1) during the 26-day experiment for the CC, CP, MC and MP treatments. Boxes show interquartile ranges, bold lines represent the median, whiskers indicate the lowest and highest values within a 1.5x interquartile range from the box, dots represent outliers. White circles and triangles represent the average concentrations in respectively container 1 and container 2.
(TIF)

S4 Fig Harvested adult Chironomus dry weight over time (a), total Chironomus biomass (larvae, adults, exuviae) (b), nitrogen (c), phosphorus (d) and carbon (e) content over time, during the 26-day experimental period. Note only treatment MC and MP are shown, since these are the only treatments harbouring Chironomus. Boxes show interquartile ranges, bold lines represent the median, whiskers indicate the lowest and highest values within a 1.5x interquartile range from the box, dots represent outliers.
(TIF)

S5 Fig Harvested Azolla dry weight over time (a), total Azolla biomass (b), nitrogen (c), phosphorus (d) and carbon (e) content over time, during the 26-day experimental period. Note only treatment CP and MP are shown, since these are the only treatments harbouring Azolla. Boxes show interquartile ranges, bold lines represent the median, whiskers indicate the lowest and highest values within a 1.5x interquartile range from the box, dots represent outliers.
(TIF)

S1 Table Results from the Dunnett T3 post-hoc test. Nitrogen, phosphorus and carbon content within the sludge is compared between treatments. t-, p-values and their significance are given. Significance (Signif.) codes: 0 ‘***’ 0.001 ‘**’ 0.01 ‘*’ 0.05 ‘.’ 0.1 ‘ ‘ 1.
(PDF)

S2 Table Elemental concentrations in different compartments of the experimental setup at the end of the 26-day experiment. (PDF)

S1 File The dataset containing all relevant data. (XLSX)
