# Supplementary figures and images for: Sludge degradation, nutrient removal and reduction of greenhouse gas emission by a Chironomus-Azolla wastewater treatment cascade
Source: PLoS One. 2024 May 28;19(5):e0301459. doi: 10.1371/journal.pone.0301459 (PMC11132448; doi:10.1371/journal.pone.0301459)

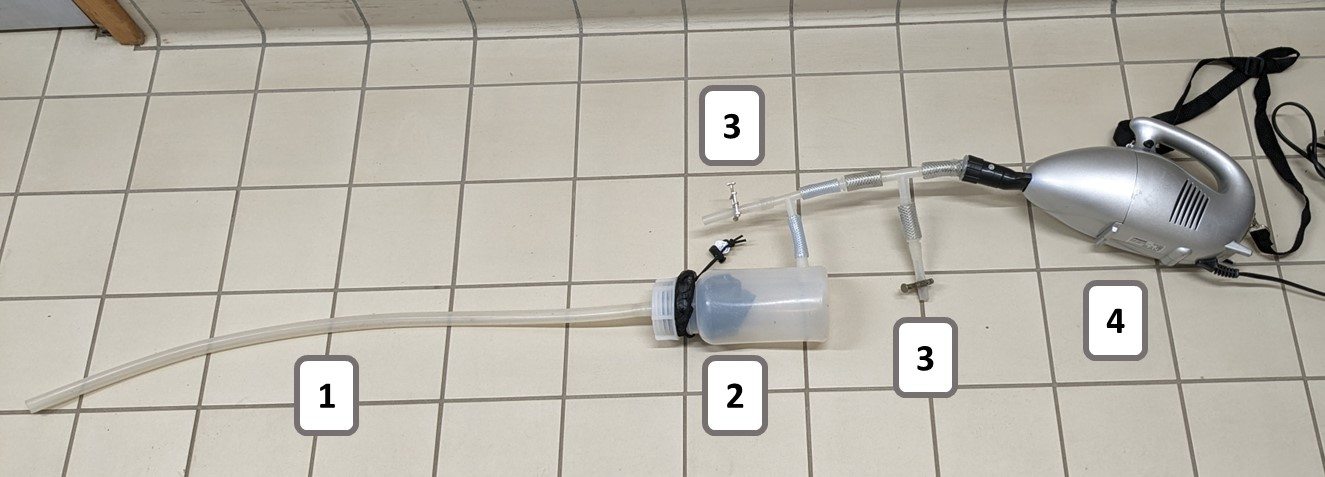

Supplement: S1 Fig — 1: Suction hose that is aimed at adult Chironomid. 2: Collection chamber and mesh bag, mesh bag can quickly be closed after vacuuming the Chironomids. 3: Tubing with valves to allow for the adjustment of suction power. 4: Vacuum device (Princess Turbotiger). (TIF) [file pone.0301459.s001.tif]

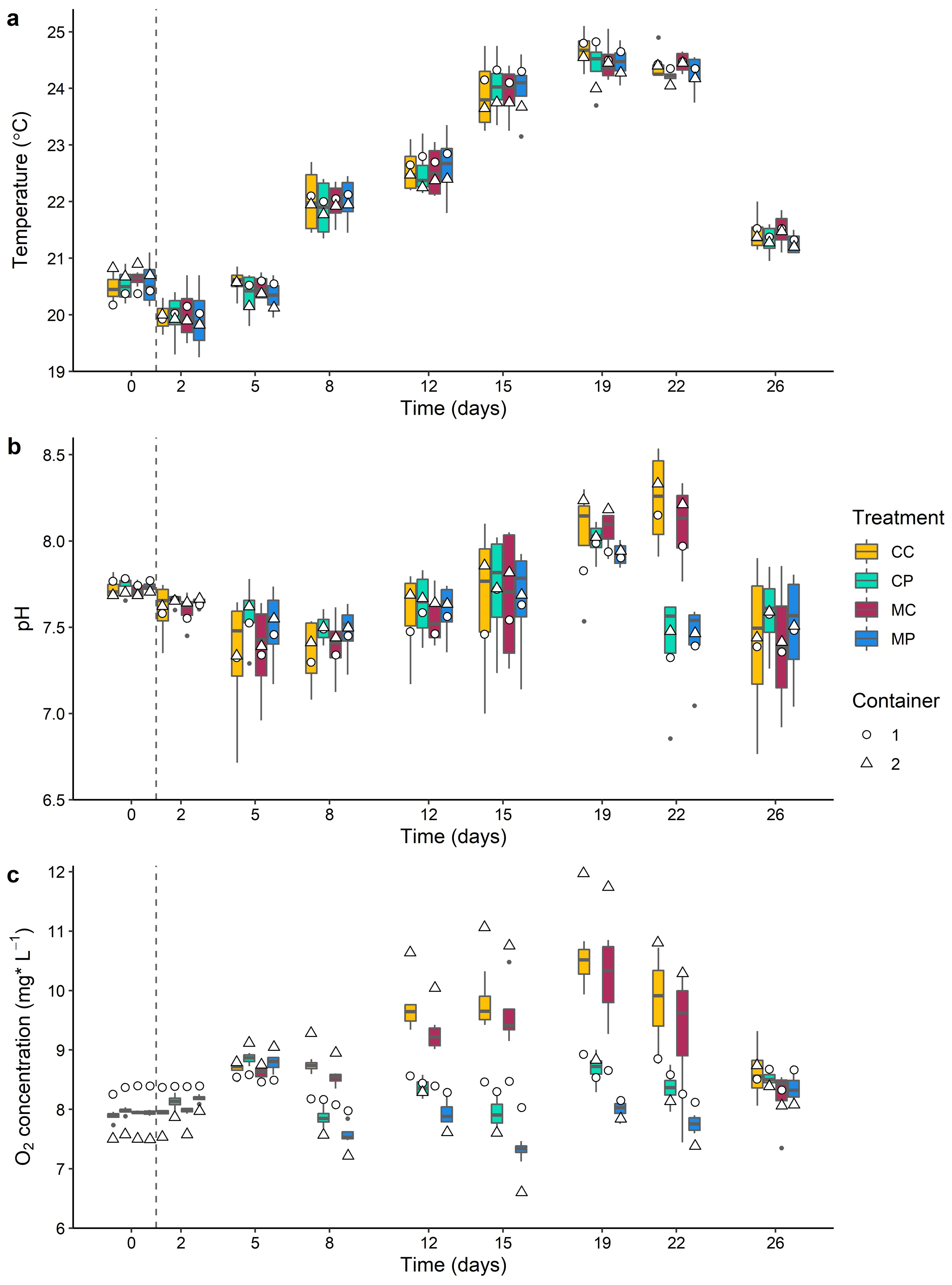

Supplement: S2 Fig — Temperature (°C) (a), pH (b) and dissolved O2 concentration (mg L-1) (c) in the overlying water during the 26-day experiment for the CC, CP, MC and MP treatments. Boxes show interquartile ranges, bold lines represent the median, whiskers indicate the lowest and highest values within a 1.5x interquartile range from the box, dots represent outliers. White circles and triangles represent the average concentrations in respectively container 1 and container 2. (TIF) [file pone.0301459.s002.tif]

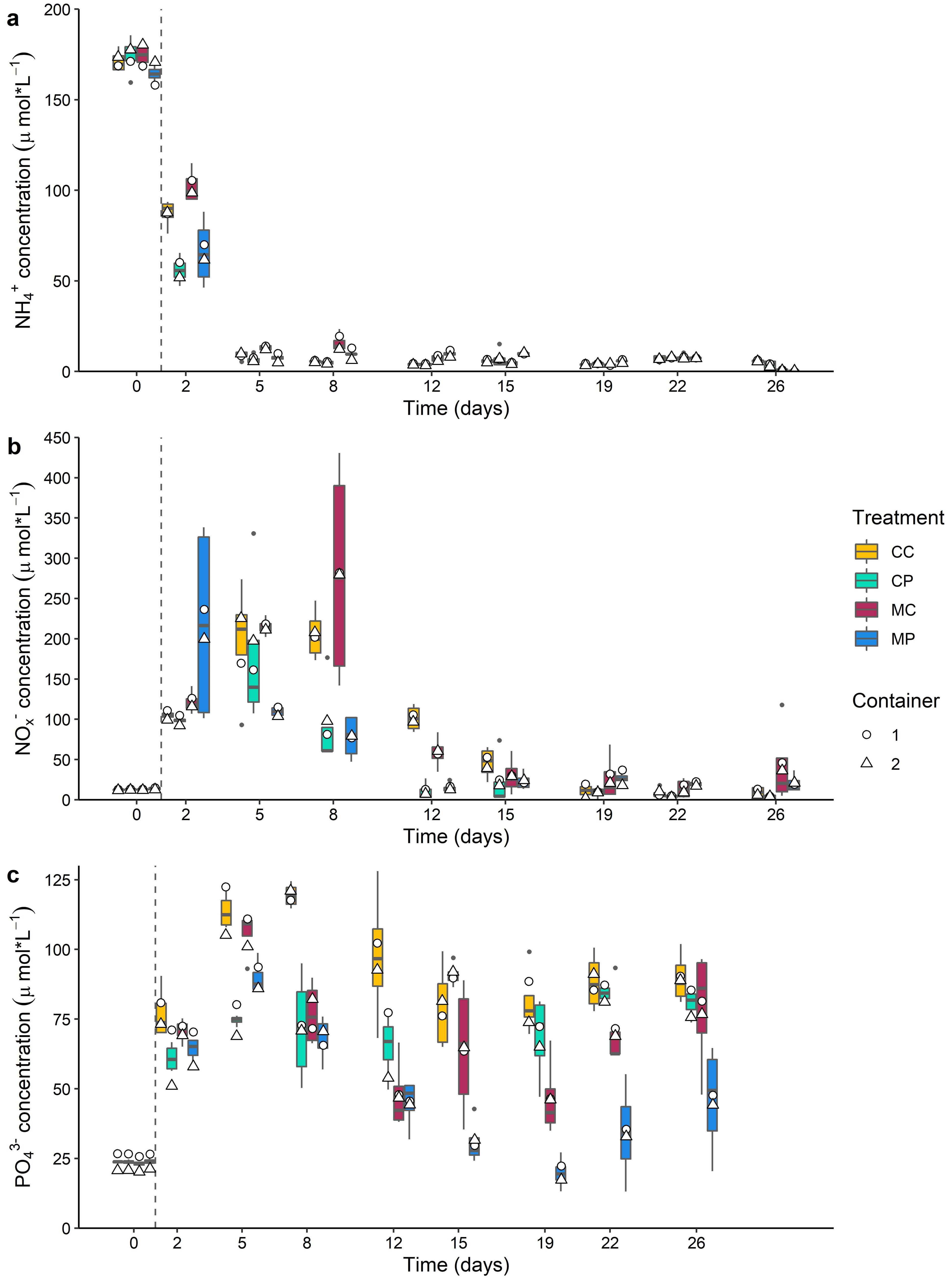

Supplement: S3 Fig — Dissolved NH4+ (a), dissolved NOx- (b) and dissolved PO43- (c) concentrations in the overlying water (μmol L-1) during the 26-day experiment for the CC, CP, MC and MP treatments. Boxes show interquartile ranges, bold lines represent the median, whiskers indicate the lowest and highest values within a 1.5x interquartile range from the box, dots represent outliers. White circles and triangles represent the average concentrations in respectively container 1 and container 2. (TIF) [file pone.0301459.s003.tif]

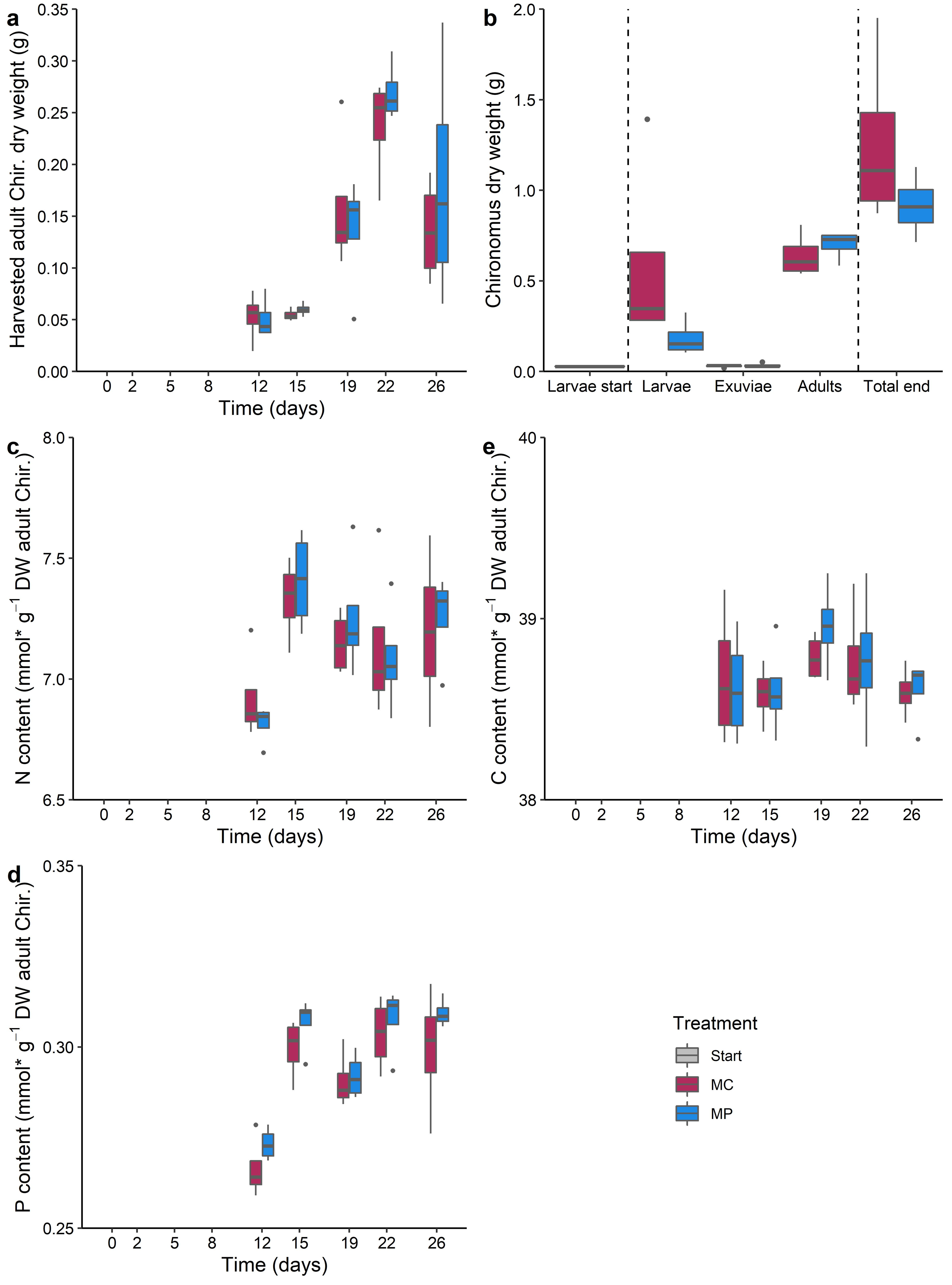

Supplement: S4 Fig — Harvested adult Chironomus dry weight over time (a), total Chironomus biomass (larvae, adults, exuviae) (b), nitrogen (c), phosphorus (d) and carbon (e) content over time, during the 26-day experimental period. Note only treatment MC and MP are shown, since these are the only treatments harbouring Chironomus. Boxes show interquartile ranges, bold lines represent the median, whiskers indicate the lowest and highest values within a 1.5x interquartile range from the box, dots represent outliers. (TIF) [file pone.0301459.s004.tif]

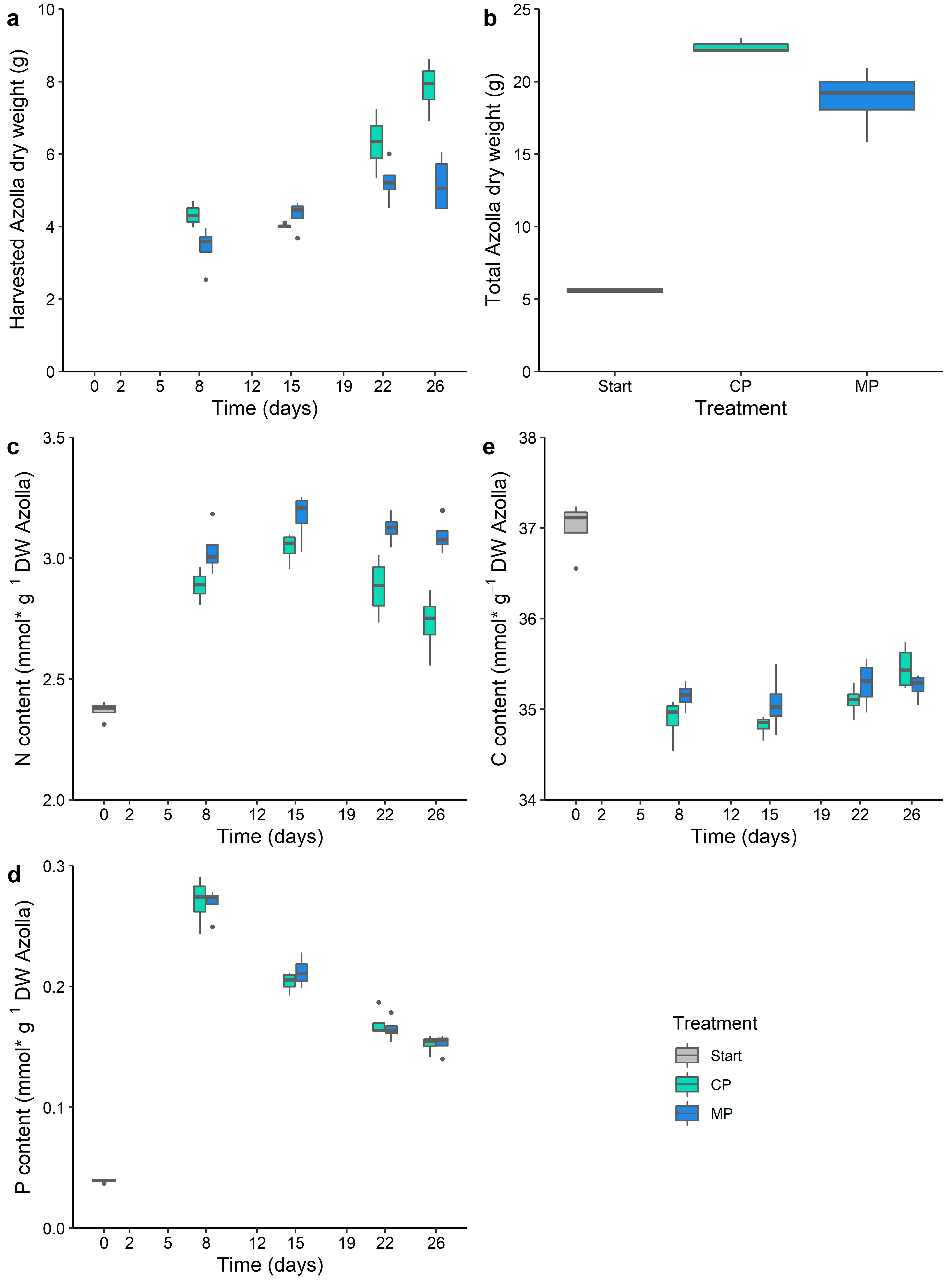

Supplement: S5 Fig — Harvested Azolla dry weight over time (a), total Azolla biomass (b), nitrogen (c), phosphorus (d) and carbon (e) content over time, during the 26-day experimental period. Note only treatment CP and MP are shown, since these are the only treatments harbouring Azolla. Boxes show interquartile ranges, bold lines represent the median, whiskers indicate the lowest and highest values within a 1.5x interquartile range from the box, dots represent outliers. (TIF) [file pone.0301459.s005.tif]
