# Supplementary material for: Sludge degradation, nutrient removal and reduction of greenhouse gas emission by a Chironomus-Azolla wastewater treatment cascade
Source: PLoS One. 2024 May 28;19(5):e0301459. doi: 10.1371/journal.pone.0301459 (PMC11132448; doi:10.1371/journal.pone.0301459)
Supplement: S1 Table — Nitrogen, phosphorus and carbon content within the sludge is compared between treatments. t-, p-values and their significance are given. Significance (Signif.) codes: 0 ‘***’ 0.001 ‘**’ 0.01 ‘*’ 0.05 ‘.’ 0.1 ‘ ‘ 1. (PDF) [file pone.0301459.s006.pdf]

S1 Table. Results from the Dunnett T3 post-hoc test. Nitrogen, phosphorus and carbon content within the sludge is compared between treatments. t-, p-values and their significance are given. Significance (Signif.) codes: 0 '***' 0.001 '**' 0.01 '*' 0.05 '.' 0.1 ' ' 1.

**Compound**				**t value**	**Pr (>|t|)**	**Signif.**
**Nitrogen**	CP	-	CC	1.838	0.4346	
	MC	-	CC	-5.103	0.0535	.
	MP	-	CC	-5.103	0.0535	.
	MC	-	CP	-5.433	0.0452	*
	MP	-	CP	-5.557	0.0425	*
	MP	-	MC	1.109	0.8263	
**Phosphorus**	CP	-	CC	-1.178	0.7905	
	MC	-	CC	4.580	0.0712	.
	MP	-	CC	3.996	0.1010	
	MC	-	CP	4.757	0.0645	.
	MP	-	CP	4.219	0.0880	.
	MP	-	MC	-1.278	0.7387	
**Carbon**	CP	-	CC	-0.017	1.0000	
	MC	-	CC	-6.068	0.0334	*
	MP	-	CC	-4.910	0.0593	.
	MC	-	CP	-5.887	0.0363	*
	MP	-	CP	-4.720	0.0385	*
	MP	-	MC	1.353	0.6961	
