# Supplementary material for: Sludge degradation, nutrient removal and reduction of greenhouse gas emission by a Chironomus-Azolla wastewater treatment cascade
Source: PLoS One. 2024 May 28;19(5):e0301459. doi: 10.1371/journal.pone.0301459 (PMC11132448; doi:10.1371/journal.pone.0301459)
Supplement: S2 Table — (PDF) [file pone.0301459.s007.pdf]

S2 Table. Elemental concentrations in different compartments of the experimental setup at the end of the 26-day experiment.

Trace element	Treatment	Mean ± SE in Sludge (µmol*g^{-1})	Mean ± SE in Water (µmol*L^{-1})	Mean ± SE in *Azolla* (µmol*g^{-1})
**Al**	CP	128.0 ± 5.3	0.61 ± 0.11	5.47 ± 0.18
	MP	159.3 ± 6.0	0.66 ± 0.12	6.14 ± 0.85
**Ca**	CP	352.1 ± 13.3	897.2 ± 14.0	196.3 ± 5.3
	MP	402.3 ± 12.8	921.1 ± 6.3	157.5 ± 1.9
**Fe**	CP	44.2 ± 1.9	0.86 ± 0.02	4.94 ± 0.41
	MP	53.9 ± 3.1	0.79 ± 0.03	3.58 ± 0.26
**K**	CP	74.0 ± 3.6	219.8 ± 12.2	1018.5 ± 44.6
	MP	164.8 ± 21.9	157.3 ± 15.1	1097.2 ± 25.7
**Mg**	CP	116.9 ± 5.6	220.9 ± 1.9	95.0 ± 2.2
	MP	229.8 ± 30.6	185.7 ± 5.2	93.7 ± 0.7
**Mn**	CP	1.13 ± 0.02	0.01 ± 0.00	0.47 ± 0.03
	MP	1.02 ± 0.02	0.00 ± 0.00	0.29 ± 0.03
**Na**	CP	151.7 ± 5.0	2657.1 ± 27.8	331.6 ± 12.8
	MP	116.2 ± 12.5	2363.4 ± 17.7	404.8 ± 18.7
**S**	CP	295.9 ± 8.2	498.6 ± 2.8	128.7 ± 7.0
	MP	283.6 ± 13.4	495.8 ± 4.9	136.5 ± 7.1
**Si**	CP	27.1 ± 1.4	2.10 ± 0.05	11.5 ± 1.9
	MP	33.7 ± 1.6	1.57 ± 0.14	5.85 ± 0.78
**Zn**	CP	13.6 ± 0.5	0.67 ± 0.04	2.04 ± 0.13
	MP	19.9 ± 1.0	0.63 ± 0.03	1.45 ± 0.07
**As**	CP	0.05 ± 0.01	0.00 ± 0.01	0.03 ± 0.02
	MP	0.04 ± 0.01	0.00 ± 0.01	0.00 ± 0.03
**B**	CP	2.41 ± 0.10	3.51 ± 0.02	2.06 ± 0.03
	MP	3.14 ± 0.14	3.49 ± 0.11	1.95 ± 0.01
**Cd**	CP	0.01 ± 0.00	0.00 ± 0.00	0.00 ± 0.00
	MP	0.01 ± 0.00	0.00 ± 0.00	0.00 ± 0.00
**Co**	CP	0.03 ± 0.00	0.02 ± 0.00	0.01 ± 0.00
	MP	0.03 ± 0.00	0.01 ± 0.00	0.01 ± 0.00
**Cr**	CP	0.29 ± 0.01	0.00 ± 0.01	0.04 ± 0.02
	MP	0.38 ± 0.02	0.00 ± 0.00	0.00 ± 0.02
**Cu**	CP	3.06 ± 0.10	0.11 ± 0.01	0.39 ± 0.01
	MP	4.37 ± 0.17	0.07 ± 0.01	0.25 ± 0.01
**Hg**	CP	0.00 ± 0.00	0.00 ± 0.00	0.00 ± 0.00
	MP	0.00 ± 0.00	0.00 ± 0.00	0.00 ± 0.00
**Mo**	CP	0.06 ± 0.00	0.00 ± 0.00	0.01 ± 0.00
	MP	0.04 ± 0.00	0.00 ± 0.00	0.01 ± 0.00
**Ni**	CP	0.23 ± 0.01	0.04 ± 0.00	0.03 ± 0.00
	MP	0.29 ± 0.01	0.04 ± 0.01	0.03 ± 0.00
**Pb**	CP	0.24 ± 0.01	0.00 ± 0.00	0.01 ± 0.00
	MP	0.30 ± 0.01	0.00 ± 0.00	0.01 ± 0.01
**Sr**	CP	0.55 ± 0.01	1.25 ± 0.01	0.33 ± 0.01
	MP	0.63 ± 0.02	1.26 ± 0.02	0.28 ± 0.01
